# Community acceptability to antimalarial mass drug administrations in Magude district, Southern Mozambique: A mixed methods study

**DOI:** 10.1371/journal.pone.0249080

**Published:** 2021-03-23

**Authors:** Beatriz Galatas, Hoticha Nhantumbo, Rodolfo Soares, Helder Djive, Ilda Murato, Wilson Simone, Eusebio Macete, N. Regina Rabinovich, Pedro Alonso, Baltazar Candrinho, Francisco Saúte, Pedro Aide, Khátia Munguambe

**Affiliations:** 1 Centro de Investigação em Saúde de Manhiça, Maputo, Mozambique; 2 ISGlobal, Hospital Clínic—Universitat de Barcelona, Barcelona, Spain; 3 Center for International Studies (CEI-IUL), Lisbon, Portugal; 4 National Directorate of Health, Ministry of Health, Maputo, Mozambique; 5 Harvard T.H. Chan School of Public Health, Boston, Massachusetts, United States of America; 6 National Malaria Control Programme (NMCP), Ministry of Health, Maputo, Mozambique; 7 National Institute of Health, Ministry of Health, Maputo, Mozambique; 8 Faculdade de Medicina, Universidade Eduardo Mondlane (UEM), Maputo, Mozambique; Instituto Rene Rachou, BRAZIL

## Abstract

**Background:**

This study aimed to capture the acceptability prior to, during and after the implementation of the first year of MDA rounds conducted under the Magude project, a malaria elimination project in southern Mozambique.

**Methods:**

This was a mixed-methods study, consisting of focus group discussions (FGDs) prior to the implementation of MDA rounds (September 2015), non-participant observations (NPOs) conducted during the MDA rounds (November 2015 –beginning of February 2016), and semi-structured interviews (SSIs) after the second round (end of February 2016). Community leaders, women in reproductive age, general members of the community, traditional healers and health professionals were recruited to capture the opinions of all representing key members of the community. A generic outline of nodes and codes was designed to analyze FGDs and SSI separately. Qualitative and quantitative NPO information was analyzed following a content analysis approach.

**Findings:**

222 participants took part in the FGDs (n = 154), and SSIs (n = 68); and 318 household visits during the MDA underwent NPOs. The community engagement campaign emerged throughout the study stages as a crucial factor for the acceptability of MDAs. Acceptability was also fostered by the community’s general will to cooperate in any government-led activity that would reduce malaria burden, the appropriate behavior and knowledge of field workers, or the fact that the intervention was available free of charge to all. Absenteeism of heads of households was identified as the main barrier for the success of the campaign. The most commonly reported factors that negatively affected acceptability were the fear of adverse events, rumors of deaths, being unable to drink alcohol while taking DHAp, or the fear to take DHAp while in anti-retroviral treatment. Pregnancy testing and malaria testing were generally well accepted by the community.

**Conclusion:**

Magude’s community generally accepted the first and second antimalarial MDA rounds, and the procedures associated to the intervention. Future implementation of antimalarial MDAs in southern Mozambique should focus on locally adapted strategies that engage the community to minimize absenteeism and refusals to the intervention.

## Introduction

The World Health Organization (WHO) currently recommends countries with standard case management and prevention tools to accelerate towards malaria elimination [1]. In this context, the use of antimalarial mass drug administration (MDA) has been revisited as a potential tool to drastically reduce transmission intensity to pre-elimination levels [2,3]. The rationale behind using MDA is to clear the parasite burden among those infected, while conferring a prophylactic effect of 2–3 months to avoid re-infections during a period of time [4].

Studies evaluating the effectiveness of MDA in different settings have revealed mixed results, with some evidence of success depending on factors such as coverage levels or adherence, both tightly linked to the community to whom the intervention was delivered [5,6]. Therefore, it is essential to ensure that antimalarial MDAs are accepted by the target communities prior to embarking in such a costly and logistically burdensome intervention [7]. Evidence of the acceptability of antimalarial MDA in Africa is scarce, but studies conducted in The Gambia and Uganda identified that strong community engagement strategies were crucial to build community’s trust and acceptability towards the intervention [8–10].

In Mozambique, the National Malaria Control Program (NMCP) has set the objective to accelerate towards malaria elimination in areas of low transmission in southern Mozambique [11]. In this context, a project known as the Magude Project was conducted in Magude district (Maputo Province), to assess the feasibility and cost-effectiveness of malaria elimination in this area [12]. The project evaluated the impact of a malaria elimination strategy based on a strengthened surveillance and case management system, intensified LLIN coverage and use, annual rounds of district-level indoor residual spraying (IRS) before the rains (August-November) followed by two monthly rounds of mass drug administration (MDA) using dihydroartemisinin-piperaquine (DHAp) for two consecutive years. The first and second MDA rounds were conducted in November 2015 and January-February of 2016, and the third and fourth rounds took place in December of 2016 and in February 2017.

MDAs were conducted door-to-door by a team of 500 local field workers and supervisors who were hired and trained in Magude by the study team in collaboration with the district authorities and community leaders. MDAs targeted the entire population of Magude and excluded per-protocol children less than 6 months old, pregnant women in the first trimester of pregnancy, and severely ill individuals [12]. Participants who were under the influence of alcohol were also excluded by the study team under the premise that they did not have adequate reasoning faculties to provide informed consent. Given that women who are pregnant in the first trimester may not be aware of their status, all consenting women of reproductive age were offered a pregnancy test before distributing DHAp. Additionally, malaria rapid diagnostic tests (RDT) were performed to all consenting MDA participants during the first round, and to the household members of a random sample of households selected throughout the district during the second round, in order to measure infection prevalence at different points in time during the implementation of the project [13]. A full dose of DHAp (once a day for three consecutive days) was provided to all consenting participants according to their age, and the first dose was directly observed by the study team. The operational MDA coverage–the number of individuals treated out of the population found in the households during the MDA visits–was between 75% and 90% for all rounds, while the effective coverage–estimated as the number of individuals treated over the population at risk of the district–ranged between 58% and 73%. Adherence to the full DHAp treatment was estimated to be between 62% and 83% depending on the assessment methodology used [13].

As part of the mix of interventions, a strong district-wide community engagement (CE) campaign was delivered in advance of MDA deployment. The messages delivered focused on informing the community about the campaign, explaining the project’s objective and importance of high levels of participation and adherence to treatment, while continuing to sleep under the bed nets and allowing IRS in their households. Although the CE campaign started in August of 2015 (3 months before the first MDA round) CE was partly informed by the findings obtained during the rapid appraisal presented in this article, that also assessed the community’s perceptions and understanding about malaria, and which results were presented elsewhere [14]. CE activities continued during the implementation of the first and second MDA, and again, before and during the third and fourth MDA. During the MDAs, the CE team focused on the rapid detection and mitigation of rumors that were identified in the communities with the aim of interrupting the expansion of misinformation that could compromise MDA coverage.

The primary aim of this study was to evaluate the community’s acceptability to the MDA rounds implemented as part of the Magude project, and generate recommendations for future MDA efforts in similar contexts. Specifically, this study aimed to rapidly assess the theoretical community acceptability and expectations to the intervention prior to its implementation; identify actual motivations and barriers for participation in the campaign and compliance to the medication; and assess acceptability and receptivity towards specific MDA procedures such as pregnancy testing among women of reproductive age (WRA) or malaria testing using RDTs.

## Methods

### Study site and population

The district of Magude is located in the north-west of Maputo province, southern Mozambique away from Mozambique’s first national road. Malaria transmission is perennial with marked seasonality between November and April, coinciding with the rainy season. There is moderate malaria transmission in the district, which experienced more than 200 cases per 1000 in the years prior to the Magude project [15]. There are 48,448 residents, and 10,965 households in the district distributed across five Administrative Posts. Magude’s population is predominantly young, half of the population does not have formal education and the main economic activities are agriculture and fishing. There are nine rural health facilities (HFs), one referral health center with an inpatient ward, and 27 community health workers (CHWs) working throughout the district [15]. CHWs provide diagnosis and treatment for malaria, diarrhea, and pneumonia, and refer patients with signs of sickness requiring higher medical attention [16]. Community sensitization about malaria has traditionally been performed in the HFs and through CHWs based on the standard social behavior change communication (SBCC) plans of the National Malaria Control Program (NMCP) [17]. Malaria control in the district has mainly consisted of standard case management using artemether-lumefantrine (AL), and vector control. The district also took part in the Lubombo Spatial Development Initiative (LSDI) which used IRS to reduce transmission in southern Mozambique between 2000 and 2011. Prior to the implementation of the Magude project, the population had barely been exposed to malaria research activities [12,15].

### Study design

This was a mixed-methods study, with a qualitative component comprised of a rapid assessment based on focus group discussions prior to the MDA rounds, and a general enquiry based on semi-structured interviews after the last MDA round of the first year of the project. The quantitative component consisted of a cross-section of structured non-participant observations conducted during the MDA rounds. Specifically, the study design followed three stages:

#### Stage 1: Rapid appraisal of acceptability prior to MDA implementation

A qualitative rapid assessment methodology was used to ensure a swift turnaround from assessment to action [18]. This approach used qualitative methods to gather the communities’ perceptions about the upcoming MDAs in order to design an appropriate CE strategy and revisit the operational aspects of the MDAs. The rapid appraisal was conducted through focus group discussions (FGDs) during the month of September of 2015, one month before the implementation of the first MDA round in November of 2015. With this assessment we aimed to understand the community’s opinions on malaria elimination as well as the *theoretical* acceptability to the procedures planned for the MDA. The latter was defined as the acceptability and predisposition towards the MDA rounds prior to their implementation. Discussions were based on a semi-structured guide of questions and were conducted under the moderation by a team of trained and experienced social science assistants, which worked in pairs of two (facilitator and observer).

FGDs were targeted to four groups of individuals with specific and homogenous characteristics, which were thought *a priori* to be representative of the community and its perspectives on MDA. The target groups included community leaders, women of reproductive age (≥ 15 years old), adult men (≥ 18 years old), and traditional healers [14]. A minimum sample of 2 FGDs with 6–12 participants per target group and administrative post were initially planned, although FGDs continued until saturation was reached. The identification of participants was performed through consultations with community key informants who acted as an intermediary between the research team and their communities.

#### Stage 2: Direct observation of participants’ reaction to MDAs, and rumor detection

Non-participant observation (NPOs) were conducted during the first and second MDA rounds (November 2015 and in January-February of 2016 respectively) in order to directly capture the actions and reactions of participants to the delivery approach, the intervention enrolment procedures, and the drug administration itself. A team of three trained observers and one supervisor accompanied randomly selected MDA field workers during the full MDA process (recruitment, evaluation of eligibility criteria, malaria and pregnancy testing and, drug administration process). A standardized structured observation guide was used to collect information and notes regarding the field worker’s and participant’s reactions throughout the observed process. Any observed event of interest associated with reasons for refusals to provide informed consent, be tested for malaria or pregnancy or being treated with DHAp were documented and investigated further through side conversations with participants after obtaining informed consent.

The target number of NPOs expected per study team per day was aligned with the target number of households established for the MDA field workers, which was an average of 4 households per day. This translated to an average of 12 NPOs per day (4 households/day x 3 observers) for approximately 15 days per MDA round (30 days for both rounds).

Simultaneously, members of the CE team who were present in the community and accompanied the MDA activities detected rumors and misinformation, and systematically reported the contents of the rumors or misinformation, source of information as well as the immediate measures taken through daily CE field reports. Reports were discussed with the supervisors and if needed with the MDA management team in order to obtain advice on how to best respond to each rumor. For the present analysis, rumor information was extracted from such reports and categorized according to the initial and emerging themes, following the content analysis approach. Rumors were defined as circulating information of an unverified account that community members heard or talked about in relation to the MDA activities during their implementation, and that could potentially interfere with individual and community trust in the intervention.

#### Stage 3: Post implementation evaluation of acceptability to the MDA campaign

Semi-structured interviews (SSIs) were conducted after the second MDA round, in February 2016. The objective of this phase was to gather participant’s reflections of the intervention after its implementation, and evaluate the *actual* acceptability to the MDA campaign based on participant’s real experiences. The main topics raised through the interviews included: 1) acceptability to the intervention; 2) motivations and barriers for participation and compliance to the medication; and 3) perceptions of the MDA campaign procedures and recommendations for improvement of coverage and acceptability rates in future MDA campaigns.

The target population for these assessments included community leaders, women of reproductive age (≥ 15 years old), adult men (≥ 18 years old), and health professionals covering the outpatient and maternity wards in all administrative areas. An initial minimum sample size of 65 SSIs was considered to be necessary to fully capture representative perspectives from all members of the community. This included a minimum of 20 community leaders, 20 women of reproductive age, 15 members of the community and 10 health workers. However, the final sample size was dictated by saturation. The same procedures used to identify FGD participants were followed during this stage.

### Data collection and analysis

**Fig 1:**
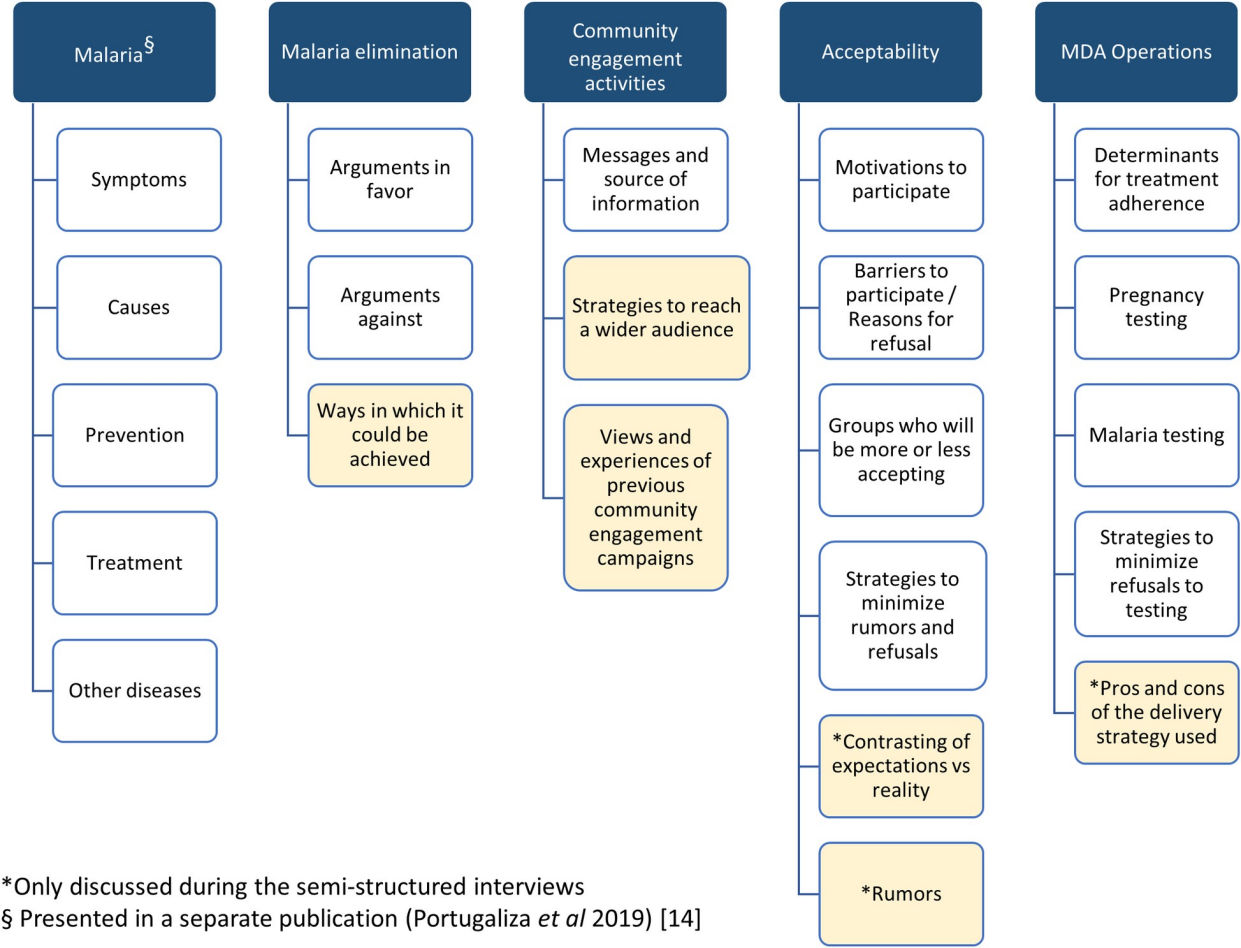
**Diagram of initial (blank) and emerging (yellow) themes and subthemes explored during the focus group discussions before the MDAs, and the semi-structured interviews after the MDAs.** Initial themes appear in blank cells while emerging themes were marked in yellow.

Semi-structured guides were designed for FGDs and SSIs in order to address their specific objectives. The guide questions were prepared in Portuguese, and pilot tested in the local language Changana. FGDs, SSIs, and NPO side conversations facilitators were fluent in both languages. Audio contents of FGDs and SSI were digitally recorded, and later on transcribed in Portuguese. Data management and analysis of FGDs and SSIs was conducted using NVivo 12 (QSR International Pty. Ltd.). A generic outline of nodes representing the coding structure was designed to analyze FGDs and SSI separately. Following a thematic analysis approach, the initial free-standing codes branched out throughout the coding process, generating coding trees which allowed including emerging themes from the development of the discussions (Fig 1). Finally, themes were critically discussed until a consensus among the researchers was reached on their relevance.

Qualitative and quantitative NPO information was collected in observation record forms and entered into an electronic data base using Redcap. Qualitative data were organized into themes that were created according to the initial and emerging themes, following the content analysis approach. With this analysis, the different responses or situations revealed by the observations were organized into categories for each theme. Finally, frequency distributions were used to summarize results obtained from the NPOs using Stata (version 14.1).

### Ethical considerations

This study was based on a protocol that was approved by CISM’s Institutional Review Board, Hospital Clínic of Barcelona’s Ethics Committee, and the Mozambican Ministry of Health National Health Bioethics Committee. All study participants had the opportunity to read or received detailed information according to what was written in a participant information sheet. Participants offered their written informed consent prior to their participation in the study. Participants anonymity and confidentiality were guaranteed throughout the research process. This was ensured by avoiding collection of participant’s full names in the study questionnaires (only their initials), and ensuring that their informed consents, study records and databases were stored in a secure server at CISM.

## Results

A total of 222 participants (154 in the FGDs and 68 in the SSIs), and 318 households participated in this mixed-methods study. FGDs and SSIs were conducted equally in all administrative posts, while the distribution of NPOs resembled that of the general population in Magude [15]. Table 1 presents the demographic characteristics of the FGD and SSI participants.

**Table 1:** Socio-demographic characteristics of the study participants of each population group of interest targeted during the focus group discussions (community leaders, men and women of reproductive age of the community [separately], and traditional healers) or the semi-structured interviews (community leaders, community members of both sexes, women of reproductive age, and health professionals).

	Focus Group Discussions (N = 154)				Semi-structured Interviews (N = 68)			
Variables	Community Leaders	Community members–Men	Community members–Women of reproductive age	Healers	Community Leaders	Community members–Men and women	Community members–Women of reproductive age	Health professionals
**Number of Participants (N)**	**52**	**39**	**53**	**10**	**20**	**20**	**20**	**8**
**Administrative post (n[%])**								
Magude sede	10 (19.2)	6 (15.4)	7 (13.2)	10 (100)	4 (20)	5 (25)	3 (15)	2 (25)
Motaze	12 (23.1)	5 (12.8)	12 (22.7)	0 (0)	4 (20)	4 (20)	4 (20)	2 (25)
Panjane	12 (23.1)	12 (30.8)	12 (22.7)	0 (0)	4 (20)	4 (20)	2 (10)	1 (12.5)
Mahele	10 (19.2)	12 (30.8)	12 (22.7)	0 (0)	4 (20)	3 (15)	7 (35)	2 (25)
Mapulanguene	8 (15.4)	4 (10.3)	10 (10.9)	0 (0)	4 (20)	4 (20)	4 (20)	1 (12.5)
**Gender (n[%])**								
Male	49 (94.2)	39 (100)	0 (0)	1 (10)	17 (85)	13 (65)	6 (75)	2 (25)
Female	3 (5.7)	0 (0)	53 (100)	9 (90)	3 (15)	7 (35)	20 (100)	0 (0)
**Marital Status (n[%])**								
Single	1 (1.9)	12 (30.8)	19 (35.8)	4 (40)	0 (0)	3 (15)	4 (20)	7 (87.5)
Married	6 (11.5)	1 (2.6)	0 (0)	1 (10)	5 (25)	2 (10)	0 (0)	0 (0)
Marital Union^1^	42 (81)	26 (66.7)	28 (51.8)	5 (50)	14 (70)	13 (65)	14 (70)	0 (0)
Widowhood	3 (5.8)	0 (0)	6 (11.3)	0 (0)	1 (5)	2 (10)	2 (10)	1 (12.5)
**Education (n[%])**								
None	13 (28.9)	2 (5.1)	11 (20.75)	1 (10)	0 (0)	7 (35)	1 (5)	0 (0)
Primary	26 (57.8)	18 (46.2)	20 (37.7)	8 (80)	14 (70)	8 (40)	15 (75)	0 (0)
Secondary	5 (11.1)	17 (43.6)	22 (41.5)	1 (10)	2 (10)	5 (25)	4 (20)	8 (100)
Higher-level	1 (2.2)	2 (5.1)	0 (0)	0 (0)	4 (20)	0 (0)	0 (0)	0 (0)
**Religion (n[%])**								
Christianity	31 (59.6)	29 (74.4)	38 (71.2)	0 (0)	19 (95)	16 (80)	17 (85)	8 (100)
Atheism	9 (17.3)	9 (23.1)	8 (15.1)	0 (0)	0 (0)	1 (5)	1 (5)	0 (0)
Animism	10 (19.2)	1 (2.6)	3 (7)	10 (100)	1 (5)	0 (0)	0 (0)	0 (0)
Islam	2 (3.8)	0 (0)	0 (0)	0 (0)	0 (0)	1 (5)	0 (0)	0 (0)
Zionism	0 (0)	0 (0)	4 (7.54)	0 (0)	0 (0)	2 (10)	2 (10)	0 (0)
**Occupation (n[%])**								
Farmer	33 (63.5)	8 (20.5.7)	29 (54.7)	0 (0)	9 (45)	11 (55)	9 (45)	0 (0)
Coal producer	0 (0)	9 (23.1)	0 (0)	0 (0)	0 (0)	0 (0)	0 (0)	0 (0)
Trad. Healer	0 (0)	0 (0)	0 (0)	10 (100)	0 (0)	0 (0)	1 (5)	0 (0)
Home-based	1 (1.9)	6 (15.4)	17 (32.1)	0 (0)	1 (5)	3 (15)	8 (40)	0 (0)
Salesperson	4 (7.7)	3 (7.7)	1 (1.9)	0 (0)	6 (30)	2 (10)	0 (0)	0 (0)
Service/Laborer	2 (3.8)	3 (7.7)	3 (5.6)	0 (0)	0 (0)	0 (0)	0 (0)	0 (0)
Driver	1 (1.9)	2 (5.1)	0 (0)	0 (0)	0 (0)	0 (0)	0 (0)	0 (0)
Teacher	1 (1.9)	1 (2.6)	0 (0)	0 (0)	1 (5)	0 (0)	0 (0)	0 (0)
Student	1 (1.9)	1 (2.6)	2 (3.8)	0 (0)	0 (0)	1 (5)	2 (10)	0 (0)
Others	10 (19.2)	6 (15.4)	1 (1.9)	0 (0)	2 (10)	3 (15)	0 (0)	8 (100)^2^

1-Marital union without marriage certificate.

2-Health professionals: district head nurse, 2 maternity nurses and 5 nurses of the outpatient wards.

### Acceptability of MDAs prior to their implementation

The rapid appraisal conducted before the first MDA round consisted of 17 FGDs, of which six were with community leaders, five with adult men, five with women of reproductive age, and one with traditional healers from the Mozambican Association of Traditional Medicine Practitioners–(AMETRAMO). The FGDs were comprised of an average of 9 participants (minimum of 3 and maximum of 12), with 2 FGDs with lower than 6 participants (one with 3 and another with 4 participants). Despite the lower number of participants, both FGDs were included in the analysis as all participants were proactive discussants and reported relevant information that reinforced the findings from the other FGDs. The distribution of socio-demographic characteristics varied per target group. Community leaders were mainly men (94.2%) while traditional healers were mainly women (90%). More than 30% of the adult men, women and traditional healers reported being single, while most community leaders reported being married or in marital union. This group had also the lowest level of formal education when compared with the adult men and women, of whom >40% reported having reached secondary education. Most of the participants were Christian and the most commonly reported occupation was farming and agriculture (Table 1).

Acceptability was queried from the stand point of the following dimensions related to malaria elimination: belief on the possibility of elimination, and views on specific components of the MDA intervention, namely community engagement, DHAp intake, and malaria and pregnancy testing.

#### Perceived possibility of malaria elimination

When questioned about the feasibility of malaria elimination, study participants generally expressed their optimism and motivation towards this possibility.

*“We don’t want it [malaria] to decline*, *we want to eliminate it because malaria kills*.*”* [FGD-04-HA-10—Adult man, Mahele].

Most respondents considered it possible to eliminate malaria. This was supported throughout the district by adult men, community leaders, traditional healers, and women of reproductive age.

*“I am sure that malaria will end*.*”* [FGD-02-HA-04—Adult man, Motaze].

However, a minority of participants doubted that malaria could be eliminated. This was particularly discussed among adult men, community leaders, and WRA in the administrative posts of Mapulanguene and Mahele. Arguments provided in support of this idea included that mosquitoes cannot be eliminated. Additionally, the notion that importation of infections from other areas would prevent the interruption of transmission was very present in participants’ discourses.

*“(*…*) we get malaria from mosquitoes*, *in order not to get malaria*, *mosquitoes have to disappear*.*”* [FGD-05-LC-14—Community leader, Mapulanguene].

*“For malaria to end here in the district*, *that is something we cannot achieve alone (*…*)”* [FGD-04-HA-10—Adult man, Mahele].

*“The disease may never end because I can leave or receive a home visit that comes from Gaza*, *coming from different districts when they have malaria*, *since some diseases are easily contagious*, *you can come visit me and discover you have malaria*, *the days you will stay*, *you can transmit it to someone in my family”* [FGD-04-HA-10—Adult man, Mahele].

*“There is no one with a medicine against the mosquito*.” [FGD-05-MIR-13 –Woman of reproductive age, Mapulanguene].

Participants suggested that in order to eliminate malaria it was necessary to eradicate mosquitoes, create a mandatory anti-malarial vaccine, or the provision of medication by the government for the prevention and treatment of malaria in the communities. Participants also highlighted the need for communities to be more conscious in their effort to prevent malaria, which they associated with receiving and using mosquito nets, accepting indoor residual spraying (IRS), taking antimalarials according to the prescription, and always keeping the house and the bathrooms clean. They also recommended that the government should be as engaged as the community in ensuring that prevention measures are being appropriately implemented and used.

*“If the government helps us with drugs and if we seriously follow the recommendations of cleaning our homes*, *building toilets*, *etc we can eliminate the disease*. *(*..*) We cannot turn our backs on the government when it wants to help*, *we have to join the government to make it feel motivated to help*”. [FGD-03-HA-07—Adult man, Panjane].

#### Views on community engagement

Study participants identified malaria health talks provided by health professionals and community leaders as fundamental to change the community’s behavior towards malaria prevention and participate in the MDA campaigns. It was also suggested that schools and churches be used as a method of disseminating health information. According to the participants, in order to secure behavior change, those who participated in community engagement meetings should share the information with other community members who did not participate in the meetings.

*“If they will accept [MDA]*, *it depends on the sensitization in each area*. *For me*, *like everyone else*, *I am concerned about malaria and I believe that the whole community will accept”* [FGD-02-HA-04—Adult man, Motaze].

*“Together we learn*. *What you’re teaching us here*, *that is not something for us to keep to ourselves*, *we must return to our place [community] and share the message to our brothers that stayed there*.*”* [FGD-02-HA-04—Adult man, Motaze].

*“All that we are talking about here*, *during our discourse on cleanings*, *on the change of mentality*, *on accepting that men enter our homes to spray them*, *this should arrive very clearly at our communities*.*”* [FGD-02-HA-04—Adult man, Motaze].

The need for a strong community engagement arose from the community’s experience to previous de-worming MDA campaigns with praziquantel that took place one month prior to the antimalarial MDAs. Participants reported neither being properly informed of the need to eat substantially before taking praziquantel, nor receiving detailed information regarding the potential side effects. To avoid rumors as a result of the side effects experienced through the de-worming campaign, community leaders recommended to engage with the neighborhood leaders (“secretaries”) who are trusted by the community.

*“We would like the community to entrust a person or perhaps the secretary*, *to come and tell the rest of us about anything*, *“x”*. *They will accept it swiftly because they trust him*, *so when the teams arrive they will have heard about it”*. [FGD-01-LC-15—Community leader, Magude Sede].

Additionally, participants reported that men working outside the country (mostly miners in South Africa) had not been well informed about events happening in the community, as they hardly ever visit their homes. This was perceived as a risk, given that the families of these men may not take part in health interventions if their household head is not present or is not well informed.

*“The main idea is to try and understand more or less … who really receives information*. *I think that our brothers working outside the country*, *especially in South Africa*, *they may think that this is not a serious intervention*.*”* [FGD-02-HA-04—Adult man, Motaze].

As a solution, community members proposed additional rounds of community engagement activities to target these men during the period when they are home during the holidays (December/January), so that they can take informed decisions, on behalf of their families, on whether or not they should participate once they are gone.

#### Theoretical acceptability to MDA

The theoretical acceptability was evaluated based on the opinions and preconceptions that participants had regarding MDA and its procedures before its implementation. In general, the community members expressed an inclination to participate in an MDA and take DHAp when the study team questioned about it.

*“Malaria is not welcome by any of us*, *in any community*. *And a sick child*, *a sick adult*, *in a home is a concern for everyone*. *A sick woman is a general concern*. *Therefore*, *everyone will accept combating malaria and trying to eliminate such a disease*.*”* [FGD-02-HAC-04—Adult man, Motaze].

Participants expected that children up to 15 years-old, women, mothers and some young adults would be the groups most likely to cooperate during the MDA rounds. This was supported by the idea that mothers and children are already familiar with taking drugs for the prevention and treatment of several diseases; and they are also the ones who most commonly seek care when they are sick.

*“With the group of the “mammas” it will be easy*, *I say that because the majority of mammas are the ones going to the hospital (*…*) they know the hospital*, *because when they go to weight their children*, *they get used to it*, *they understand*, *it is easier to take medication”* [FGD-05-LC-14—Community leader, Mapulanguene].

**Fig 2:**
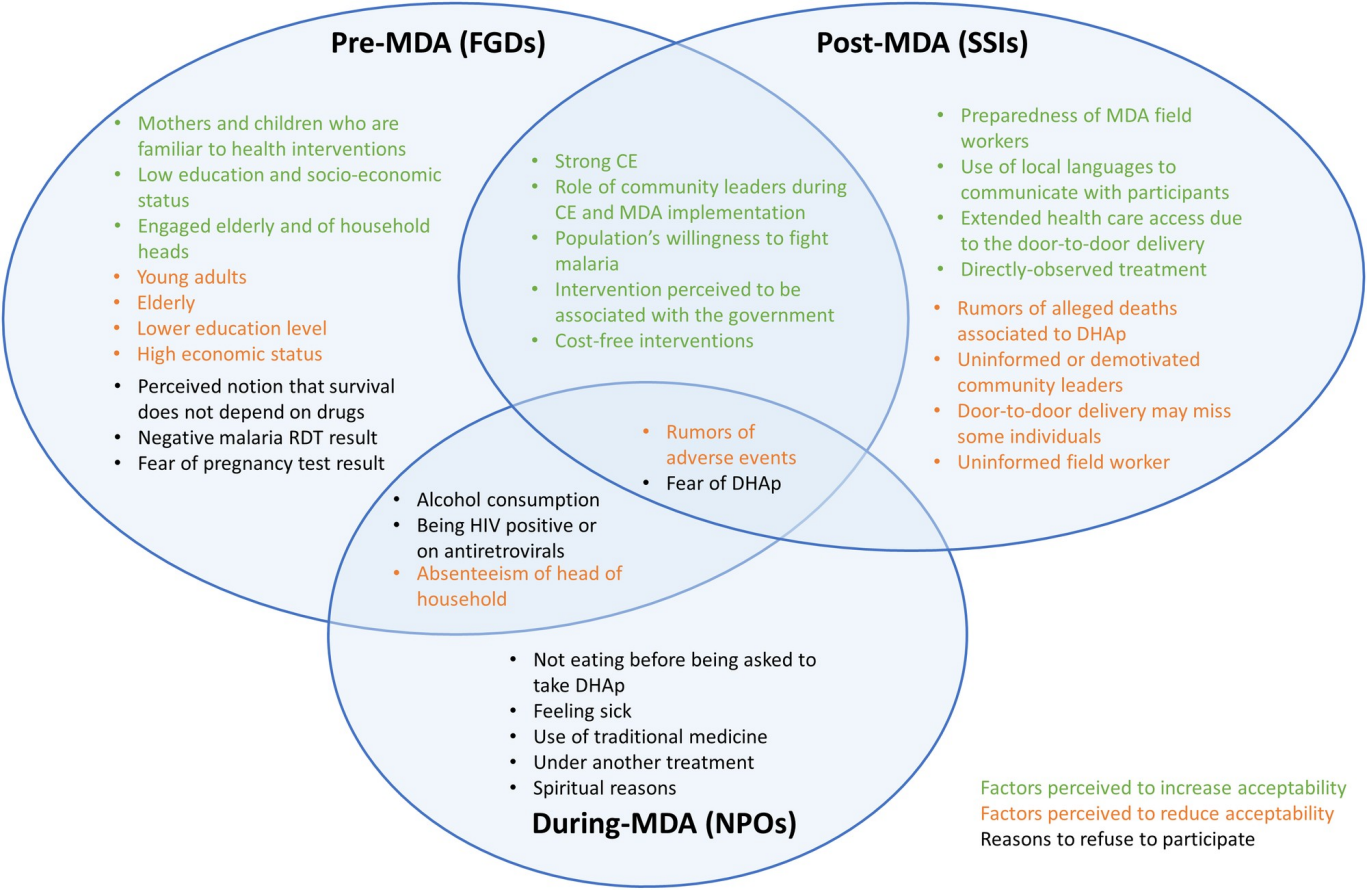
Factors reported by study participants before, during and after MDA implementation, that were perceived affect acceptability through focus group discussions (FGDs), non-participant observations (NPOs) or semi-structured interviews (SSIs), respectively. Factors perceived to increase acceptability are marked in green and those believed to decrease acceptability appear in orange. Potential reasons for refusal identified before the intervention, observed during the intervention and reported after the intervention are presented in black.

When asked about the population groups who would potentially be more resistant to the intervention, a generational confrontation occurred between younger and older participants. The elderly identified young individuals as the most likely to refuse to participate, for a variety of reasons including that they will not be able to drink alcohol for three consecutive days while on treatment. They also identified factors such as being irresponsible, stubborn or uninformed, as risk factors that would affect their acceptance and participation in the campaign. Interestingly, these characteristics were also used by the younger participants to describe the elderly when arguing that they would be the least accepting group (Fig 2).

*“When there is something happening in the area*, *the hardest ones are usually the boys*. *Even when there are meetings with young people*, *they will not show up*. *Even the [de-worming] campaign we had in the hospital*, *they didn’t go*.*”* [FGD-04-MIR-12—Woman of reproductive age, Magude Sede].

*“I hear what the old man says*, *but the elderly are far more complicated and they will refuse*. *I heard some of them saying*, *"I can no longer take those pills*, *they came with some pills to kill us*, *we got here [grew up] without the pills*, *we don’t live off pills"*. *Us*, *young people*, *we know we will take the pills*, *there is no one who will refuse*, *but I hear older people saying they will not take them “they want to give me pills to kill me”*. *Old men will refuse*, *younger people will accept*.” [FGD-03-HAC-07—Adult man, Panjane].

However, discussions with different study groups across the district consistently identified that younger individuals would be more likely to accept MDAs, given their higher education levels compared to the elderly. Participants explained that older individuals are generally less likely to seek care, and are therefore more resilient to taking medications.

*“In the past we lived with those medicines*, *from those trees that you are looking at [refers to traditional medicine]*, *now they increase our diseases through these things*, *and old people are the most difficult*, *once they realize that they need to go to the hospital*, *they refuse to do it [refers to complying with hospital rules]*.*”* [FGD-05-LC-14—Community leader, Mapulanguene].

The association between formal education and willingness to take part in the MDA rounds was evident in the discussions; and participants believed that those less educated would be less likely to understand the messages passed on to them through engagement campaigns, and would consequently be more reluctant to participate in MDAs. However, assuming those with lower education attainment also have a low socio-economic status, participants believed that this group of people would be more likely to accept MDAs given that the antimalarial drugs are distributed for free. They also believed that small businessmen or individuals of higher economic status would be less likely to participate given that they can pay for medical assistance and therefore, will not be interested in free drugs.

*“The ones that create difficulties are the powerful (*…*) the ones saying that you can buy pills somewhere else*, *in the big clinics*, *these are the ones creating problems*. *They won’t even attend the meetings*. *With older people and people of average economic conditions there will be no problems*. *But the ones that will bring us problems are those small businessmen*, *more here (Magude Sede)*, *in the countryside they will accept*.*”* [FGD-01-LC-01—Community leader, Magude Sede].

Other reasons for refusal to the MDA verbalized by the community included the suspicion of the government’s intention to sterilize the population in order to control the HIV epidemic in the area, by which they believe to be highly affected in the district, or to reduce the population size. The fear of being used to test the efficacy of a new drug, with the risk of suffering from unknown adverse effects, was also vocalized as a potential reason for refusal.

*“Because if another person other than the secretary arrives*, *there will be so many rumors—"ah*, *they already want to kill*, *they are tired of us because there is AIDS and they realized they can’t end it*. “[FGD-01-LC-15—Community leader, Magude Sede].

*“…they may want to reduce our numbers using tricks here in Mozambique*, *because we are too many*, *we have to be reduced*. *Some people think like this*.*”* [FGD-01-LC-15—Community leader, Magude Sede].

*“These pills we have to take have not been tested*, *they want to test in us*. *What if they bring negative effects*? *They come and test*… *so I’m afraid we’ll try it without knowing its effects*.*”* [FGD-04-HA-10—Adult man, Mahele].

As a solution to the potential refusals, which was an emerging theme from the discussions, the community members recommended engaging the elderly to inform them of the lack of repercussions of the medication on their health. Adult male heads of household were also identified as key players for the acceptance of MDA, given their role as decision-makers in their households. Some community leaders suggested that the government should apply a penalty to those who refuse to participate in such campaigns, arguing that their negative actions rend the whole effort useless.

*“When you do not want to take the tablets*, *it’s your life that is being thrown away*, *because the government gave us tablets to take (*…*) the government makes an effort to fight malaria*, *provides tablets*, *if you throw them away*, *it is your fault*, *you are the one that is going to die”* [FGD-03-LC-08—Community leader, Panjane].

*“The government should enforce a punishment on the person that is hard to convince*, *imagine if some refuses to collaborate*, *what will the [health] workers do*? *It’s hard…”* [FGD-03-LC-08—Community leader, Panjane].

#### Theoretical acceptability to pregnancy testing

Women of reproductive age were grateful for the explanation provided to them during the community engagement campaigns about the need for pregnancy testing (PT), and communicated that they were aware that, if pregnant, they could only take the drug after the first trimester.

*“We are grateful to those that came to our houses [to inform us]*, *door to door*, *as otherwise we would all flee*, *myself*, *I would run away to hide [during the MDAs]”*[FGD-04-MIR-12—Woman of reproductive age, Mahele].

Several reasons for accepting or refusing to take a pregnancy test during the MDAs were revealed when participants were questioned about this procedure. Single and married women (or in marital union) who want to have children were expected to accept the pregnancy test as this was an opportunity for them to check their status.

However, participants also identified a number of barriers to the acceptance of the PT. First, younger women may find a way of being absent on the day of the MDA rounds in their area, in fear of detecting an unplanned pregnancy and of the consequences linked to parental disapproval. On the other hand, a rejection to undertake the test would be seen by the parents as suspicious, therefore creating tensions between parents and daughters.

*“Here you will have war*, *why won’t you take the test*? *People will even fight*, *why don’t you want to take the test*?*”* [FGD-01-MT-17—Traditional healer, Magude Sede].

Second, some married women may refuse to take a PT, in fear that her husband may distrust her for not yet having told her, or accuse her of adultery in the case of a positive result. Finally, women may refuse to take a PT in fear of having to inform their mother-in-laws, especially if their husbands are in South Africa.

To minimize the refusals to PTs, participants recommended communicating to women that the PT will be performed in private and that the result will be kept confidential. They also deemed fundamental to provide clear information about the need for the pregnancy test within the campaign.

#### Theoretical acceptability to malaria testing with RDTs

Participants communicated that they were aware that the intake of DHAp was not conditional on the malaria test result, and that the community would take the drug, even in the case of a negative RDT. To support this claim, they referred to previous MDA experiences in which drugs were taken despite not being ill. Additionally, the RDT procedures were not seen as a problem given the populations’ experience with vaccines and other blood tests.

“*Will you accept the needle prick without any problem*? *[Simultaneous voices] They will prick us*. *If its children they are used to it because at the hospital they get vaccines*, *at school they get vaccines*, *and when we get sick we are pricked*.*”* [FGD-01-MIR-06—Women of reproductive age, Magude Sede].

Nevertheless, several barriers associated to malaria testing also emerged. Some women mentioned that an RDT-negative result would likely affect the individual’s motivation to take DHAp, even after receiving the explanation that the drug also protects against future infections. Other reasons for refusing to undertake an RDT included that some individuals may simply not want to be tested, while others will not want their families/the field worker to know about their results, or may not want to know about their own results. Malaria testing was also feared as some associated it with the discovery of additional diseases or HIV status.

*“In testing of malaria you may find other diseases (*…*) Because*, *whenever one is examined something is found*. *Anemia*, *and many things*.*”* [FGD-04-LC-11—Community leader, Mahele].

*“People don’t like to be tested and know their health*, *some are afraid of catching HIV*, *others are afraid of not healing*, *others don’t want people to know they have a disease or that they are on medication*. *We die because of this*, *fear of the test*.*”*[FGD-02-LC-03—Community leader, Motaze].

Participants emphasized the importance of communicating that testing will only be done for malaria, and that RDT results will be treated with confidentiality.

### Participants’ reactions to MDAs during implementation, and detected rumors

A total of 318 NPOs were performed during household visits where 1166 individuals were found. Of these, 164 (51.6%) NPOs took place during the first MDA round (November 2015), including 577 individuals (49.5%); and 154 (48.4%) NPOs took place during the second MDA round (January-February 2016), involving 589 (50.5%) participants. An average of 11 NPOs were conducted per day (3.7 per observer per day). Out of all household visits performed, 107 heads of households (33.7%) were absent during the visit. The main reasons for this included not being at home during the day (36%), being at work (19%), or emigrating outside of the district for a medium/long term period of time (29%). In these households, heads of households were substituted by their wives (70.1%), their daughters in law (9.4%) or their siblings (6.5%).

In terms of observed acceptance to receive the MDA field team, only one of the observed visited household rejected the entrance of MDA field workers, given the absenteeism of the head of the household, and the unwillingness of his wife to make any decisions on his behalf. There were 53 (16.7%) heads of households who raised questions to the field workers during the informed consent phase. Participants questioned field workers about the potential adverse events of DHAp (30.2%) as well as the effects of the drugs taken with alcohol (13.2%), on an empty stomach (9.4%) or in combination with antiretroviral treatment (11.3%), anti-tuberculosis treatment (1.9%) or traditional medicine (1.9%). Some participants wondered why this study was conducted in Magude (3.7%), the rational for treating uninfected individuals (5.7%) or whether they could participate in the second round given that they did not participate in the first one (3.7%) (Table 2).

**Table 2:** NPO participants questions during the MDA, and reported reasons for refusing to accept a malaria RDT, pregnancy test, or DHAp.

Questions raised by participants (N = 53)	n	%
DHAP intake with other treatments	1	1.9
DHAp intake with TB treatment	1	1.9
DHAp intake with Traditional medicine	1	1.9
DHAp intake if s/he had malaria recently	1	1.9
Sexual relationships while taking DHAp	1	1.9
Reasons for asking for a pregnancy test	1	1.9
Reason for study being done in Magude	2	3.8
Ability to participate in MDA2 if did not participate in MDA1	2	3.8
Purpose of DHAp	2	3.8
Other	2	3.8
DHAp prophylaxis duration	2	3.8
Adverse events associated with deworming campaign	3	5.7
Reasons for treating the uninfected	3	5.7
Adverse events in absence of food	5	9.4
DHAp with ARVs	6	11.3
Alcohol intake while on DHAp	7	13.2
Adverse events	13	24.5
**Reasons of malaria RDT refusal (N = 16)**		
Has malaria	1	6.3
Refused	1	6.3
Is a traditional healer	1	6.3
Tradition or spiritual reasons	3	6.3
Baby (<1-year-old)	3	18.8
Scared of the RDT	3	18.8
Alcohol	4	25.0
**Reasons of pregnancy test refusal (N = 58)**		
Had already been tested during MDA1	1	1.7
On birth control	1	1.7
Could not urinate	1	1.7
Has malaria	1	1.7
Not sexually active	1	1.7
Under other treatment	1	1.7
Widow	1	1.7
Not fertile	2	3.5
Has a baby	3	5.2
Her husband is not home	3	5.2
Menstruating	3	5.2
Refused	5	8.6
Breastfeeding	13	22.4
She is not pregnant	22	37.9
**Reasons of DHAp refusal (N = 76)**		
The mother refused	1	1.3
The mother was not home	1	1.3
No trust in DHAp	1	1.3
Spiritual reasons	1	1.3
Traditional healer	1	1.3
Does not take pills	1	1.3
Had malaria recently	3	3.9
On traditional medicine	4	5.3
Is sick	5	6.6
Under another medication	5	6.6
HIV-positive	6	7.9
Refused	7	9.2
Had not yet eaten	9	11.8
Adverse events	11	14.5
Alcohol intake	20	26.3

After the informed consent phase, there were 16 (5%) NPOs where RDT testing was refused. The main reasons provided involved being under the influence of alcohol (4) being less than a year old (3), being scared of finger-pricking (3), or due to spiritual or traditional reasons (3). One traditional healer refused to be tested, and one individual had already been diagnosed with malaria in the hospital and was on antimalarial treatment (Table 2).

There were 58 (18.3%) NPOs where at least one woman of reproductive age refused to take a pregnancy test. The most common reasons for refusal included knowing that they were not pregnant (37.9%), having a small baby and/or breastfeeding (27.6%). Other women reported being menstruating (5.2%), or being unable to take a pregnancy test in their husband’s absence (5.2%). Additional reasons for refusal included being on birth control or unfertile; or being sexually inactive or widowed (Table 2).

Refusals to accept DHAp treatment were observed in 76 (23.9%) NPOs. The most common reason for refusal was alcohol intake (26.3%), followed by fear of adverse events (14.5%) or not having eaten by the time of the visit (11.8%). Being HIV positive, or anti-retroviral treatment (ARVs) (7.9%), feeling sick (6.6%), or taking traditional medicine (5.3%) were alternative reasons for not accepting DHAp treatment (Table 2).

During the MDAs, the CE team recorded 20 instances of rumors, which were responded to during the CE sessions. Half of the rumors were detected in the administrative post of Magude Sede (the most urbanized and populated area of the district), and a few others were detected in Motaze, Panjane and Mapulanguene administrative posts, or through the MDA hotline made available during the activities for Magude residents to communicate any concerns or adverse events during the intervention implementation. Rumors of deaths associated to DHAp were the most reported by the CE team (11/20). Three of these rumors referred to two specific persons: a health professional reported hearing that a female 18 year-old had died (possibly referring to the SAE that took place during MDA1); and several households in the same neighborhood of Magude Sede reported hearing that an “important” woman, or the wife of an “important person” in the community had died due to the DHAp. This report triggered an investigation by the study team, which identified that the woman had not participated in the study. This information was fed back to community members whenever it was opportune. Other rumors of deaths were non-specific:

“*People are saying that the drug kills”* [Vendors of the Magude Sede Market].

Other detected rumors were about the adverse events of DHAp, and adverse events or deaths from praziquantel (7/20). A pregnant woman reported hearing that DHAp could cause abortions, and an SMS was sent to the hotline from an unknown person who reported hearing about CISM’s research activities in Manhiça district, where malaria was still detected, and demanded that CISM did not expand its activities to Magude. The CE team responded to all rumors through targeted meetings and messages, involving key players of the communities where rumors were detected.

### Acceptability to MDA after its implementation

Semi-structured interviews were conducted with community leaders (20), members of the community (20), women of reproductive age (20), and health professionals (8). The latter group included the district head nurse, 2 maternity nurses and 5 nurses of the outpatient wards. The socio-demographic characteristics distribution of community leaders, women and members of the community were similar to that observed among FGD participants (Table 1).

Participants’ experiences and reflections of the MDA rounds were captured through the following dimensions: views on the intervention, perceived motivations and barriers for participation and compliance to the medication; and perceptions of the MDA procedures including the delivery method, and the approach to malaria and pregnancy testing.

#### Post-implementation reflections on acceptability to MDA

Semi-structured interviewees generally reported that the MDAs were well accepted by the community.

*“The population was happy with this work”* [SSI-03-MIR-12—Woman of reproductive age, Panjane].

*“For what I saw and heard*, *the population was grateful for this intervention that will prevent us from falling sick from malaria”* [SSI-02-LC-11—Community leader, Motaze].

There are several factors that study participants identified as key for the high level of acceptability perceived during the MDAs. First, the participation of community leaders (political, administrative, traditional or religious) and neighborhood secretaries during the community engagement activities as well as during the MDAs provided role model figures for the wider the community. Second, they reported that information was clearly provided to them during the community engagement activities as well as by the MDA field workers. However, some reported that community engagement activities were not carried out homogeneously, and certain neighborhood secretaries were reported not being fully committed to the campaign and not communicating with the communities on a regular basis, which resulted in distrust and strong resistance (Fig 2).

*“I think it was because of poor explanations*. *And here we can say that some secretaries may have not performed well the sensitization*, *because if that* [community adherence] *happened in one neighborhood*, *why not in the others*?*”* [SSI-02-LC-10—Community leader, Motaze].

Additional aspects that positively influenced acceptability of the campaign according to SSI participants included the cost-free nature of the treatment, the preparedness of the MDA field workers, population’s willingness to fight malaria and the fact that local languages were used in the community engagement activities.

According to interviewees, acceptability was affected by rumors associated with the occurrence of adverse events and alleged deaths perceived to have occurred during the MDAs potentially related or unrelated to DHAp intake. These rumors spread around central Magude causing apprehension in some communities.

*“The challenges faced were related to the drugs delivered during the first round*, *as they caused diarrea*, *while in other places I heard that people died after taking the drugs*, *that’s what I heard; so they were worried of taking them in the second round*. *However*, *as we were sensitized*, *we continued to recommend to take the drug”* [SSI-01-LC-11 –Community leader, Magude Sede].

*“The big noise came when my mother-in-law passed away [*…*]*, *that rumor was meant to create doubt and jeopardize the work*, *while in truth when we went to her burial*, *we were informed that she was sick*, *no one blamed CISM*, *she took no pills*, *she died of a prolonged disease*.*”* [SSI-01-LC-14—Community leader, Magude Sede].

Contrary to what was captured during the theoretical acceptability assessment, alcohol consumption, widely perceived as potential reason for refusal to take DHAp prior to its implementation, was not reported as a major element of non-participation according to interviewees, even during the second MDA round, which coincided with the *canhú* season (a wild fruit with which a traditional alcoholic beverage of the same name is produced). One participant declared that even if the population knew DHAp should not be taken with alcohol, some participants would hide that they were drunk in order to take DHAp.

*“Even the second time [round]*, *it was coincident with the canhú season*, *they [population] said “eh*! *If we drink we’ll be afraid [of taking DHAp]*, *but they took it nonetheless”* [SSI-02-MC-14—Community member, Motaze].

Health professionals were responsible for monitoring adverse effects of the medication in the population. One of them reported that they would have liked to be more involved in the campaign activities, particularly to share information and experiences with MDA field workers. Health professionals also complained about stock-outs of medications used for alleviating the secondary effects of DHAp.

*“We must review the management of the drugs to manage adverse events*. *We tried as much as possible to control our stock*, *but it was not enough*. *Sometimes people show up unexpectedly and we cannot [help them]*. *I appeal to other districts*, *to control and properly manage the drugs that prevent adverse events*.*”* [SSI-01-PS-02—Health professional, Magude Sede].

#### Post implementation views on MDA procedures

Part of the community approved and appreciated the door-to-door delivery strategy used for the MDA rounds, while others were reticent about it. Those who supported the door-to-door strategy argued that it extended the access to health care to less privileged community members, as well as to those who do not seek care at the health facilities. Others preferred it given that they did not have to queue at the hospital.

*“They [campaign fieldworkers] entered many houses; only a careless person would have not taken them [the pills] (…) Its possible*, *because it avoids one being at a queue at the hospital*, *it will avoid crowds in there because of disease*” [SSI-03-MC-03—Community member, Panjane].

However, some community leaders mentioned that the door-to-door distribution of antimalarials surprised their communities, as they were used to receiving drugs at the hospital. Some also thought that this delivery strategy negatively impacted coverage, given that those who refused to participate simply left their houses on the day of the visit. Similarly, participants reported that this strategy affected coverage given the absenteeism of several household heads during the visit days, which translated to non-participation of all present household members.

*“There were different points of view*, *because we people have a way of being*, *you can find a group saying "I’ve never seen medication being distributed from house to house*, *could it be they want to kill us*? *Why are they not distributed at the hospital*?*"*, *so I*, *as the secretary*, *convened a meeting and said “gentlemen*, *the government cannot allow this project to leave*… *it is government money*, *because the government want us*, *their people*, *[they want us] to be healthy*. *That is why we value the effort of the government that got a project to give us pills in our homes*, *instead of going to the hospital to suffer*.*”* [SSI-01-LC-14—Community leader, Magude Sede].

Most of the interviewees did not complain about taste, color or size of the DHAp tablets. They considered them to be standard, like any other tablet. The dosage regimen was also considered acceptable. Study participants generally accepted the directly observed treatment (DOT) of the first dose of DHAp. One health professional believed that some members of the community would have otherwise not taken the medication on their own, based on her experience with tuberculosis patients.

*“For example*, *I have seen this from the part of tuberculosis patients*, *when they are given medicine to take at home*, *they do not take it*. *Now*, *for example*, *"in neighborhood 2"*, *they would come here in the morning*, *I would give water and the pill*, *they take and leave*. *That’s very good*. *You can give them the pills*, *they arrive at their home and they store it*. *It is important to watch*, *see the person taking it*.*”* [ESE-05-PS-08—Health professional, Mapulanguene].

Participants also reported being suspicious of field workers when they did not correctly follow the procedures that were expected of them, as a result of the information communicated during the community engagement campaigns. Some reported that MDA field workers provided wrong dosages of DHAp, while others were worried that they did not bring scales with them in order to dose individuals according to their weight. Community members also criticized field workers who did not warn about adverse events, or who failed to mention that DHAp needs to be taken with food, despite the latter being contrary to the recommendations for DHAp intake.

“*They were afraid of the dosage because they are quite strong and also because it was heard that a scale would be used*, *however when they* [field workers] *arrived they only used their eyes to make a reading*, *they would look and say “he is around 70 kg” and they would give it [DHAp]”* [SSI-01-LC-14—Community leader, Magude Sede].

In any case, MDA field worker’s work ethics and attitude during their interactions with the communities were highly praised. Participants described them as patient, and polite; and appreciated their good behavior when someone refused to take DHAp. In those cases, they reported that field workers provided clear information and returned the following day, allowing time for household members to ponder on their decision.

*“They worked with love*, *they displayed respect*, *we are thankful*.*”* [SSI-05-LC-09—Community leader, Mapulanguene].

*“It went well*, *because upon arrival and if you refused*, *they* [field workers] *would ask you to take it [DHAp]*, *they would not force you to take it*. *They would even say if you don’t want it we will return*, *and they would return the next day until we would take it”* [SSI-04-MIR-10—Woman of reproductive age, Mahele].

Participants recommended that MDA field workers should always follow procedures when performing their duties. They also recommended to adapt the campaign procedures to the local context, such as addressing each household and household member in a culturally appropriate way, identifying where to perform the MDA tasks, or knowing how to behave when inside someone’s house. Participants also recommended that MDA workers should have previous fieldwork experience.

#### Revealing experiences of pregnancy testing after implementation

The attitude of women’s parents and husbands towards the pregnancy test was mostly positive, allowing and supporting their wives and daughters to participate. However, interviewees reported that in many cases women would not agree to be tested, given that they already knew their pregnancy status. Additionally, some parents did not encourage their young daughters to be tested, especially in the case of girls who had recently had their menarche, claiming that they were still children. Thus, the expected issues with acceptability towards the pregnancy test reported during the FGDs were not observed during the MDAs, as a community leader from Panjane reported.

*“I had an opinion* [previously to MDA rounds], *but I changed my mind*. *What made me change was*, *at first I thought it* [the PT] *would cause fuss*, *why*? *Because they had to test women to know if they are pregnant whilst at* [the women’s] *home this pregnancy is not known*, *but then we saw that it went well*, *because we had no problems*, *so I had to change my initial opinion*. *There was no trouble*, *even in the houses we visited; there was no difficulty in performing the test*. *I thought it would be difficult but I didn’t hear anything complicated*. *I doubted before* [the MDA] *but now that they did it*, *there were no difficulties”* [SSI-03-LC-05—Community leader, Panjane].

#### Revealing experiences of malaria testing after implementation

The majority of interviewees appreciated being tested for malaria during the MDAs and they reported being aware of the procedures, which were described as "pricking the finger". Participants generally reported having been informed that DHAp could serve as a treatment or as prevention against malaria, and accepted to take the drug despite a negative RDT result. Nevertheless, some interviewees mentioned that only testing MDA participants for malaria in the first MDA round but not in the second caused uneasiness in the community. This was supported with the argument that they would rather be tested and take the drugs in spite of a negative result, than not being tested at all.

“*The important thing*, *what they should not do is to come with pills and give it to us without testing our blood to know the diseases we have*, *whether its malaria or not*, *it has to be seen*” [SSI-02-LC-12—Woman of reproductive age, Motaze].

“*Receiving pills without being tested created some discomfort among us*” [SSI-03-LC-12—Community Leader, administrative post].

The above revelation of experiences also suggests that community members might not be completely aware that malaria RDTs only serve to test for malaria and not other infections or diseases.

## Discussion

This three-stage mixed-methods study revealed that the antimalarial MDAs that took place in Magude as part of the malaria elimination project were generally well accepted by a community with previous exposure to similar interventions targeting other neglected tropical diseases. This was observed in spite of the belief among some participants that malaria elimination could not be achieved. The community engagement campaign implemented in Magude, which took into consideration and attempted to address local communities’ concerns and expectations, emerged as a crucial factor for the acceptability of MDAs throughout all study phases. In line with other MDA efforts [6,19,20], this suggests that adequate messages that local communities relate to are key to catalyze the integration of new conceptual elements into the participants’ cognitive universe, whilst fostering stronger community involvement and health literacy [21,22]. Other factors that positively affected MDA acceptability included the community’s will to cooperate in any government-initiated activity that would reduce malaria burden, the appropriate behavior and knowledge of field workers when performing the household visits, or the fact that they viewed the MDAs as a nationally-led intervention available free of charge to all.

The most commonly reported factor that negatively affected acceptability during all stages of the study was the fear of adverse events, driven by the recent experience with the de-worming MDA campaign that took place one month before the first MDA round in Magude. While the district’s pharmacovigilance system recorded adverse events such as headache, vomiting or asthenia during the MDAs, these were reported in very low numbers [13]. Thus, it is possible that such fears were driven and potentially sustained by rumors which were also linked to the suspicion of MDA being an experimental exercise. Additionally, the upsurge of rumors of deaths associated to DHAp intake, was identified as a factor that affected MDA acceptability during its implementation. While a death possibly associated to DHAp intake was indeed detected during the first MDA round [13], most of the rumors detected in this socio-behavioral study arose from several alleged deaths that occurred during the MDA period according to people’s perceptions. Although the rumors were not directly captured by the NPOs, they were also picked-up by the community engagement team during the MDA campaigns, who continuously reacted to dissipate them and manage misinformation on this topic. Overall, perceptions regarding adverse events and death rumors may have been exacerbated by the nature of the project, which was done under an individual-level informed consent that included information on potential adverse events, with a special emphasis on pharmacovigilance. Thus, such negatively affecting factors may be less prevalent when MDAs are performed in programmatic mode.

Aligned with what had been anticipated by the FGD participants before the MDAs, being under the influence of alcohol was reported as one of the major reasons of no participation in the MDA rounds during the NPOs. This was particularly observed during the second MDA round that coincided with the season of the traditional drink *canhú*, either due to the lack of capacity to provide informed consent, or because participants wanted to be able to drink in the future [23]. Interestingly, alcohol consumption or drunkenness were not considered by SSI interviewees as major risk factors or reasons reported by the community to not participate in the MDA rounds. In fact, SSI interviewees reported anecdotes of individuals who did not disclaim to the MDA field workers that they had taken *canhú* in order to take DHAp. The anecdotes communicated by SSI interviewees reveal the challenges associated with large-scale trials conducted under informed consent in the community. In our setting, field workers were instructed to ensure that all MDA participants had adequate reasoning faculties to provide informed consent before recruiting them to participate in the MDA activities. As a result, some individuals who were evidently under the influence of alcohol were either excluded by field workers or simply refused to participate. This resulted in a lower MDA coverage despite there being no contraindications of DHAp intake and alcohol consumption. Overall, these findings are relevant in contexts where alcohol use is prevalent in the community, as health interventions need to be designed in ways that maximize intervention coverage while guaranteeing participants’ safety and voluntariness.

The above mentioned factors were reported in most stages of the study. However, there were also substantial differences between the anticipated factors and actual reasons for refusal that could affect acceptability, compared to what was observed or reported during or after the implementation of MDA (Fig 2). Age, gender, education level, and socio-economic status were reported by FGD participants as key factors expected to negatively or positively affect acceptability. Nevertheless, participants of the post-MDA campaign SSIs reported, based on their experience with the campaign, that the preparedness of field workers, use and good command of local languages, rumors of death or low motivated community leaders were some of the main aspects that they perceived to have affected MDA participation. FGD participants also expected community members to refuse participation under the premise that they do not need medication to survive, or that they do not have malaria (specially with a negative malaria RDT result). Such findings were not supported by the NPOs or SSIs. This is also observed through the experience of intermittent preventative treatment during pregnancy (IPTp), another antimalarial-drug intervention implemented in Magude aiming to prevent malaria infections in pregnant women. In fact, the coverage of IPTp among the MDA-participating pregnant women in the second or third trimester was above 90% among the ~70% who reported attending the ante-natal clinics [13]. This suggests that, at least among this group, the women of Magude are accepting of this type of interventions. In any case, as raised during the FGDs, the concepts around malaria chemoprevention should always be reinforced when elaborating messages associated to drug-based interventions aiming for prevention.

Another perceived aspect that FGD participants expected to affect MDA participation was the fear of the detection of an unwanted or unknown pregnancy. However, evidence collected during the NPOs and SSIs did not support the expectations of FGD participants. In fact, malaria and pregnancy testing were also considerably less challenging in practice than as expected. Information collected through the NPOs revealed that the majority of refusals to pregnancy testing were driven by women’s certainty of their pregnancy status, rather than by the fear of revealing a pregnancy to family members. This is of specific relevance among adolescent women, whose attitudes were not particularly explored in this study, and for whom further research should focus on.

Such contradiction between the FGD and NPO or SSI findings exposes the limitations of rapid acceptability assessments prior to the deployment of a new intervention, as individuals’ opinions may be influenced by pre-conceptions and overly expectations which may lead to incomplete conclusions with regards to acceptability [24]. Nonetheless, the information obtained throughout the FGDs was crucial to obtain initial insights into community’s concerns and expectations, which in turn were important for the design and adaptation of the messages and target population of the community engagement campaign, key for the success of the intervention [14]. Above all, from a research perspective, the triangulation of pre- and post- implementation data on community expectations and experiences revealed important socio demographic and contextual factors identified by the community members themselves, such as age, education, socio-economic or migration status. These are worth considering when generating and testing hypothesis and conceptual frameworks on factors influencing MDA acceptability.

From an implementation perspective, this study also revealed local drivers of coverage and uptake that should be considered if antimalarial MDAs are to be planned and rolled out with success in southern Mozambique. First, the absenteeism of many heads of households in the community was identified as a major limitation for coverage reported by study participants prior and during the MDAs was. The main reasons for absence observed during the NPOs included not being at home during the day, or emigrating outside of the district for a medium/long term period of time. The latter is likely driven by the high emigration rate of southern Mozambican men to South Africa, which was commonly reported in the FGDs [25]. During the observed visits 33.7% of household heads were absent, and were substituted by another present member. Thus, MDA delivery mechanisms that consider a high likelihood of household head absenteeism and plan to minimize its impact on coverage will likely achieve a higher impact associated to the intervention. In this context, the door-to-door approach was discussed by various SSI interviewees. Some believed this delivery strategy improved coverage as it expanded the access to care to communities that do not usually seek care, either because of their socio-economic status or because they do not want to “suffer in the hospital”. The latter reason is particularly relevant as it suggests that quality of care at the health facility may be perceived to be low, and the community members prefer being treated at home. On the other hand, SSI participants also reported that the door-to-door approach could miss individuals who deliberately left their homes during the MDA visit because they did not want to participate.

Second, the high burden of HIV that affects Magude, as well as other neighboring areas of southern Mozambique [26] also emerged as a key contextual aspect to factor in when planning antimalarial MDAs in this area. This was largely driven by two arguments. First, the skepticism that the MDA rounds were intended to diagnose, or even kill HIV-infected individuals. Through the discussion of the linkages between HIV status and acceptance of malaria RDT it transpired that malaria infection may be subject to some level of stigma in this community. Study participants recommended that confidentiality of the malaria test results be observed so that individuals who tested positive and their families are protected from social harm. This was exacerbated by the limited knowledge about the specificity of RDT to malaria. The second argument for the association of HIV prevalence and MDA acceptability was the fear of taking additional drugs for prevention of future malaria infections while on anti-retroviral treatment. This is a relevant aspect to consider when implementing MDAs with DHAp in areas with similarly high levels of HIV transmission, given the contraindications of DHAp with antibiotic or antifungal treatments.

Several recommendations emerged from the discussions before and after the MDA to minimize the aspects that affect acceptability or potential reasons to refuse participation. As suggested by participants, this requires a thorough community inquiry into their contextual factors, alongside strong, flexible and continuous community engagement efforts [20,27]. This should be done in close collaboration with adequate community members and local authorities, and considering the needs of specific population groups. Some of the most important groups highlighted by study participants include men who live away from their households for long periods of time, community leaders and the elderly. Some of these lessons were incorporated to the community engagement activities of the third and fourth MDA rounds of the Magude project (not covered through this qualitative assessment), and the individual-level data collected during the MDAs revealed that the number of individuals who were eligible but refused to participate decreased between the first and second, and the third and fourth MDA rounds [13]. It is possible that the improved community engagement activities, the increasing exposure to MDAs or a combination of the two could explain the reduction in refusals through time. Unfortunately, the second year of MDAs were not accompanied by a qualitative assessment that allowed responding to these questions.

The results obtained from this study need to be interpreted considering the following limitations. First, the community engagement activities planned before and during this assessment could have shaped the community’s views and acceptability to antimalarial MDAs. Second, this study was conducted before the outcome of the project was known. While very drastic reductions in the incidence and prevalence of malaria were recorded in Magude as a result of the project, malaria transmission was not interrupted, a finding that was similarly observed in other countries in Africa [13,28,29]. The observed acceptability to MDAs may have differed if this result was known. However, evidence of the substantial levels of acceptance to mass administrations of drugs for neglected tropical diseases, despite of their adverse events [30], as well as to other malaria preventative tools, suggest that antimalarial MDAs may still be acceptable to the communities despite not leading to the full interruption of the disease. Third, findings are based on self-reported information, both based on future expectations and recalled experience. However, drawing respondents from across different population groups and across the community, as well as including direct and non-participant observation as data collection techniques, and collecting data at different stages of the MDAs implementation, allowed to triangulate the results and minimize potential bias arising from a single perspective of specific population groups, specific study periods (before, during or after the intervention) or data collection approaches. Fourth, desirability bias could have affected the reported information among participants in the FGDs—associated with higher skepticism towards a new intervention, and SSIs—driven by the desire to please the interviewers who were seen to be associated to the intervention, or by the expectation of extra benefits from the future interventions. Finally, the transcription and translation process and the deployment of multiple interviewers could have led to the potential loss of some information. Nevertheless, interviewers were trained to implement the study, transcribe and analyze the information homogeneously, and results were validated by in-house/local researchers.

## Conclusion

This study revealed that the community of Magude generally accepted the MDA rounds that took place during the first year of the Magude project, and were in conformity with the procedures associated to the intervention, albeit punctual conceptual discomfort with certain approaches such as rapid testing for malaria or pregnancy. The initial skepticism towards this new type of antimalarial intervention revealed by the FGDs, was replaced by a general sense of acceptance driven by empirical experience. Acceptability was mainly driven by the intensified community engagement campaigns constantly adapted to the local context, and a delivery strategy that promoted community ownership and trust. Fear of adverse events or deaths, arose as the major reasons for refusal to take DHAp among householders. However, the largest barrier for high MDA coverage was the absenteeism of key household members and certain community representatives during the household visits, which likely affected the overall impact of the malaria elimination project. Future implementation of antimalarial MDAs in these areas should focus on locally adapted information strategies that engage the community as a whole, tailoring for specific groups likely to be sidelined, to minimize refusals and absenteeism and maximize the intervention’s impact.

## Abbreviations

ARV: Anti-retroviral treatment
CE: Community Engagement
DHAp: Dihydroartemisinin Piperaquine
FGDs: Focus Group Discussions
HIV: Human Immunodeficiency Virus
IRS: Indoor Residual Spraying
IEC: Information Education Communication
MDA: Massive drug administration
NPOs: Non-participant Observations
RDT: Rapid diagnostic test
SSI: Semi-structured Interviews
WRA: Women of Reproductive Age

## References

[1] World Health Organization, Global Malaria Programme. Global technical strategy for malaria, 2016–2030 [Internet]. 2015. Available from: https://www.who.int/malaria/areas/global_technical_strategy/en/.

[2] von Seidlein L, Greenwood BM. Mass administrations of antimalarial drugs. Trends in Parasitology. 2003;19:452–60. 10.1016/j.pt.2003.08.003 14519583

[3] Eisele TP. Mass drug administration can be a valuable addition to the malaria elimination toolbox. Malar J. 2019;18:281. 10.1186/s12936-019-2906-8 31438950 PMC6704699

[4] World Health Organization. Mass drug administration for falciparum malaria: a practical field manual. Geneva: World Health Organization. 2017.

[5] Poirot E, Skarbinski J, Sinclair D, Kachur SP, Slutsker L, Hwang J. Mass drug administration for malaria. Cochrane Infectious Diseases Group, editor. Cochrane Database of Systematic Reviews. 2013;12:CD009946. 10.1002/14651858.CD008846.pub2 24318836 PMC4468927

[6] Newby G, Hwang J, Koita K, Chen I, Greenwood B, von Seidlein L, et al. Review of Mass Drug Administration for Malaria and Its Operational Challenges. American Journal of Tropical Medicine and Hygiene. 2015;93:125–34. 10.4269/ajtmh.14-0254 26013371 PMC4497884

[7] Pell C, Tripura R, Nguon C, Cheah P, Davoeung C, Heng C, et al. Mass anti-malarial administration in western Cambodia: a qualitative study of factors affecting coverage. Malaria Journal [Internet]. 2017 [cited 2017 May 22];16. Available from: http://malariajournal.biomedcentral.com/articles/10.1186/s12936-017-1854-4. 10.1186/s12936-017-1854-4 28526019 PMC5438518

[8] De Martin S, Von Seidlein L, Deen JL, Pinder M, Walraven G, Greenwood B. Community perceptions of a mass administration of an antimalarial drug combination in The Gambia. Tropical Medicine & International Health. 2001;6:442–8. 10.1046/j.1365-3156.2001.00723.x 11422958

[9] Wanzira H, Naiga S, Mulebeke R, Bukenya F, Nabukenya M, Omoding O, et al. Community facilitators and barriers to a successful implementation of mass drug administration and indoor residual spraying for malaria prevention in Uganda: a qualitative study. Malaria Journal. 2018;17:474. 10.1186/s12936-018-2624-7 30558632 PMC6298012

[10] Jaiteh F, Masunaga Y, Okebe J, D’Alessandro U, Balen J, Bradley J, et al. Community perspectives on treating asymptomatic infections for malaria elimination in The Gambia. Malaria Journal. 2019;18:39. 10.1186/s12936-019-2672-7 30777112 PMC6378745

[11] National Malaria Control Program (NMCP), Ministerio da Saúde (MISAU). Plano Estratégico de Malária 2017–2022, Moçambique. MISAU, NMCP; 2017.

[12] Aide P, Candrinho B, Galatas B, Munguambe K, Guinovart C, Luis F, et al. Setting the scene and generating evidence for malaria elimination in Southern Mozambique. Malaria Journal. 2019;18:190. 10.1186/s12936-019-2832-9 31170984 PMC6554892

[13] Galatas B, Saúte F, Martí-Soler H, Guinovart C, Nhamussua L, Simone W, et al. A multiphase program for malaria elimination in southern Mozambique (the Magude project): A before-after study. PLOS Medicine. Public Library of Science; 2020;17:e1003227. 10.1371/journal.pmed.1003227 32797101 PMC7428052

[14] Portugaliza HP, Galatas B, Nhantumbo H, Djive H, Murato I, Saúte F, et al. Examining community perceptions of malaria to inform elimination efforts in Southern Mozambique: a qualitative study. Malaria Journal [Internet]. 2019 [cited 2019 Aug 21];18. Available from: https://malariajournal.biomedcentral.com/articles/10.1186/s12936-019-2867-y. 10.1186/s12936-019-2867-y 31296238 PMC6625114

[15] Galatas B, Nhacolo A, Martí-Soler H, Munguambe H, Jamise E, Guinovart C, et al. Demographic and health community-based surveys to inform a malaria elimination project in Magude district, southern Mozambique. BMJ Open. 2020;10:e033985. 10.1136/bmjopen-2019-033985 32371510 PMC7228537

[16] Ndima SD, Sidat M, Give C, Ormel H, Kok MC, Taegtmeyer M. Supervision of community health workers in Mozambique: a qualitative study of factors influencing motivation and programme implementation. Human Resources for Health. 2015;13:63. 10.1186/s12960-015-0063-x 26323970 PMC4556309

[17] Arroz JAH. Social and behavior change communication in the fight against malaria in Mozambique. Rev Saude Publica. 2017;51:18. 10.1590/S1518-8787.2017051006360 28355338 PMC5342323

[18] Guise A, Witzel TC, Mandal S, Sabin C, Rhodes T, Nardone A, et al. A qualitative assessment of the acceptability of hepatitis C remote self-testing and self-sampling amongst people who use drugs in London, UK. BMC Infect Dis. 2018;18:281. 10.1186/s12879-018-3185-7 29914381 PMC6006927

[19] Njomo DW, Mukoko DA, Nyamongo NK, Karanja J. Increasing Coverage in Mass Drug Administration for Lymphatic Filariasis Elimination in an Urban Setting: a Study of Malindi Town, Kenya. Shiff C, editor. PLoS ONE. 2014;9:e83413. 10.1371/journal.pone.0083413 24454703 PMC3891599

[20] Pell C, Tripura R, Nguon C, Cheah P, Davoeung C, Heng C, et al. Mass anti-malarial administration in western Cambodia: a qualitative study of factors affecting coverage. Malaria Journal. 2017;16:206. 10.1186/s12936-017-1854-4 28526019 PMC5438518

[21] Nguyen T-N, Thu PNH, Hung NT, Son DH, Tien NT, Van Dung N, et al. Community perceptions of targeted anti-malarial mass drug administrations in two provinces in Vietnam: a quantitative survey. Malaria Journal [Internet]. 2017 [cited 2017 May 22];16. Available from: http://malariajournal.biomedcentral.com/articles/10.1186/s12936-016-1662-2. 10.1186/s12936-016-1651-5 28061908 PMC5216593

[22] Sturrock HJ, Hsiang MS, Cohen JM, Smith DL, Greenhouse B, Bousema T, et al. Targeting asymptomatic malaria infections: active surveillance in control and elimination. 2013 [cited 2015 Jul 1]; Available from: http://dx.plos.org/10.1371/journal.pmed.1001467. 10.1371/journal.pmed.1001467 23853551 PMC3708701

[23] Magaia T, da Cruz Francisco J, Uamusse A, Sjöholm I, Skog K. EDIBLE WILD FRUITS OF MOZAMBIQUE. Acta Hortic. 2012;223–8.

[24] Sekhon M, Cartwright M, Francis JJ. Acceptability of healthcare interventions: an overview of reviews and development of a theoretical framework. BMC Health Services Research. 2017;17:88. 10.1186/s12913-017-2031-8 28126032 PMC5267473

[25] Nhacolo AQ, Nhalungo DA, Sacoor CN, Aponte JJ, Thompson R, Alonso P. Levels and trends of demographic indices in southern rural Mozambique: evidence from demographic surveillance in Manhiça district. BMC Public Health [Internet]. 2006 [cited 2017 Jun 6];6. Available from: http://bmcpublichealth.biomedcentral.com/articles/10.1186/1471-2458-6-291. 10.1186/1471-2458-6-291 PMC1712340 17137494

[26] González R, Augusto OJ, Munguambe K, Pierrat C, Pedro EN, Sacoor C, et al. HIV Incidence and Spatial Clustering in a Rural Area of Southern Mozambique. PLoS ONE. 2015;10:e0132053. 10.1371/journal.pone.0132053 26147473 PMC4493140

[27] Kajeechiwa L, Thwin MM, Shee PW, Yee NL, Elvina E, Peapah P, et al. The acceptability of mass administrations of anti-malarial drugs as part of targeted malaria elimination in villages along the Thai–Myanmar border. Malaria Journal [Internet]. 2016 [cited 2017 May 22];15. Available from: http://malariajournal.biomedcentral.com/articles/10.1186/s12936-016-1528-7. 10.1186/s12936-016-1528-7 27677694 PMC5039796

[28] Daniels RF, Schaffner SF, Bennett A, Porter TR, Yukich JO, Mulube C, et al. Evidence for Reduced Malaria Parasite Population after Application of Population-Level Antimalarial Drug Strategies in Southern Province, Zambia. The American Journal of Tropical Medicine and Hygiene; 2020;tpmd190666. 10.4269/ajtmh.19-0666 32618255 PMC7416975

[29] Deng C, Huang B, Wang Q, Wu W, Zheng S, Zhang H, et al. Large-scale Artemisinin-Piperaquine Mass Drug Administration With or Without Primaquine Dramatically Reduces Malaria in a Highly Endemic Region of Africa. Clin Infect Dis. 2018;67:1670–6. 10.1093/cid/ciy364 29846536 PMC6455902

[30] Kisoka WJ, Tersbøl BP, Meyrowitsch DW, Simonsen PE, Mushi DL. Community members’ perceptions of mass drug administration for control of lymphatic filariasis in rural and urban Tanzania. J Biosoc Sci. 2016;48:94–112. 10.1017/S0021932015000024 25790081 PMC4668335

